# Supplementary figures and images for: Dynactin has two antagonistic regulatory domains and exerts opposing effects on dynein motility
Source: PLoS One. 2017 Aug 29;12(8):e0183672. doi: 10.1371/journal.pone.0183672 (PMC5574551; doi:10.1371/journal.pone.0183672)

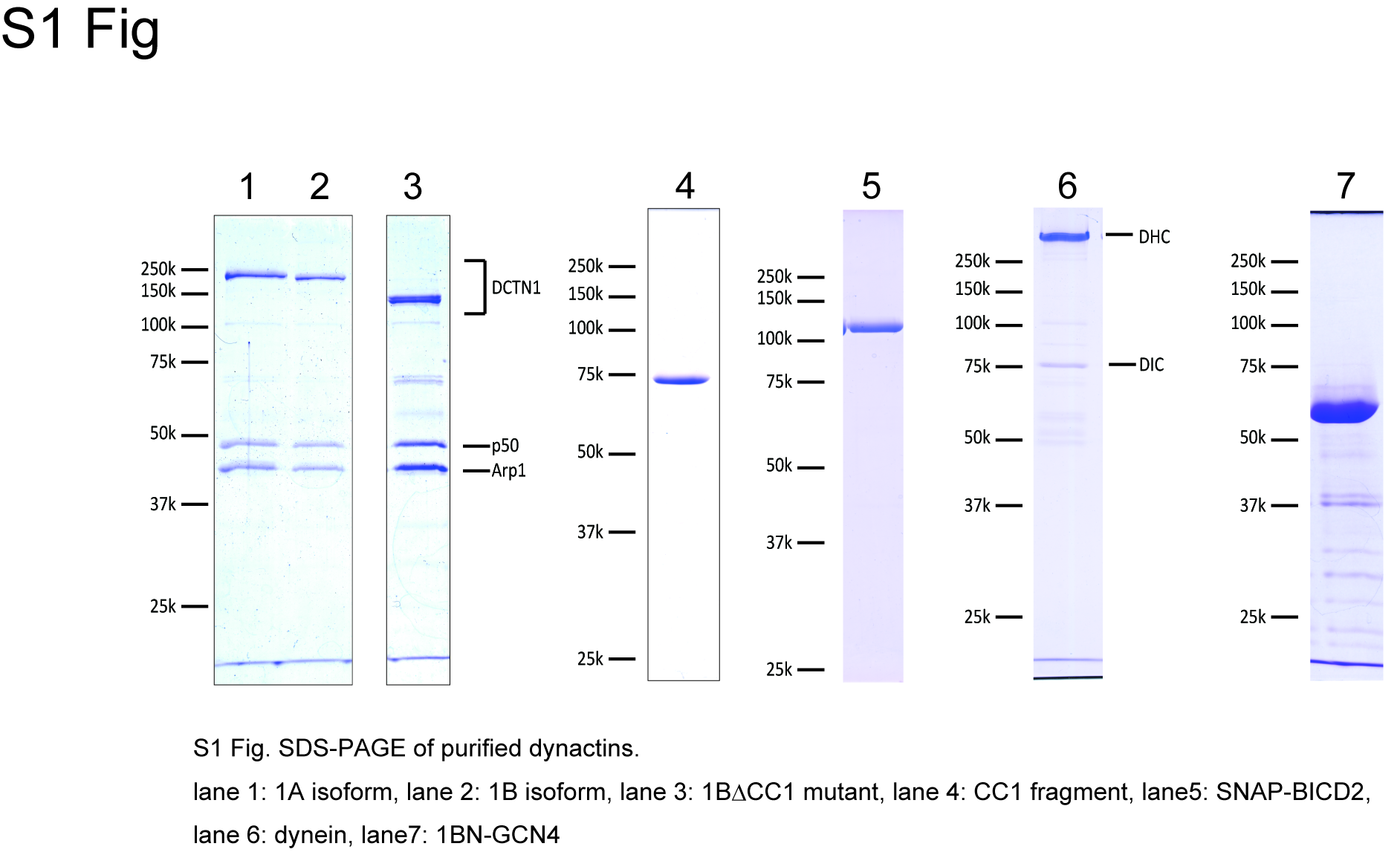

Supplement: S1 Fig — Lane 1: marker; lane 2: 1A isoform; lane 3: 1B isoform; lane 3: 1BΔCC1 mutant, lane 4: CC1 fragment, lane 5: SNAP-BICD2, lane 6: dynein, lane 7: 1BN-GCN4. (TIF) [file pone.0183672.s001.tif]

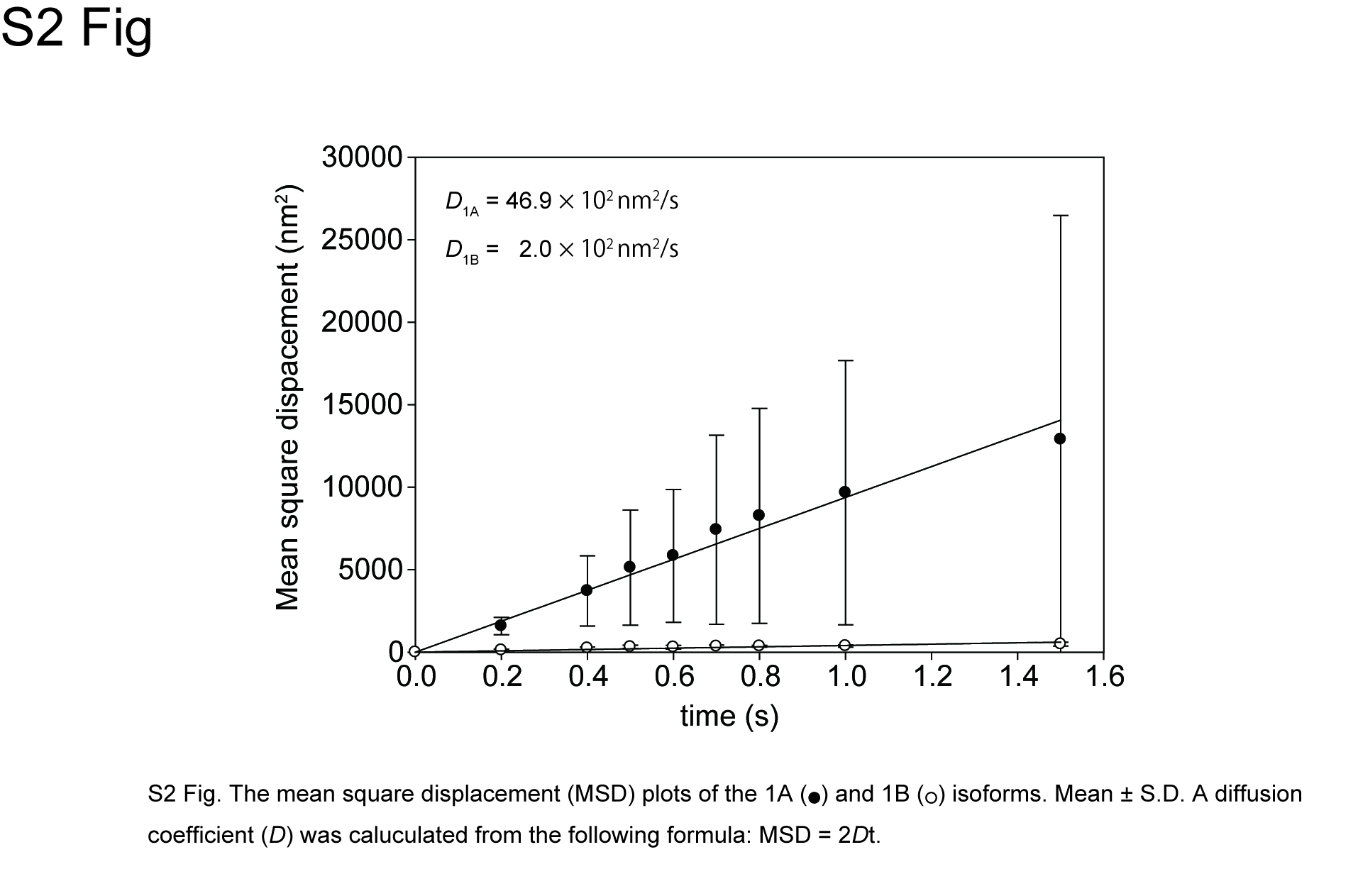

Supplement: S2 Fig — MSD plots of 1A (●) and 1B (○) isoforms. Mean ± S.D. The diffusion coefficient (D) was calculated by the following formula: MSD = 2Dt. (TIF) [file pone.0183672.s002.tif]
