# Supplementary material for: Dynactin has two antagonistic regulatory domains and exerts opposing effects on dynein motility
Source: PLoS One. 2017 Aug 29;12(8):e0183672. doi: 10.1371/journal.pone.0183672 (PMC5574551; doi:10.1371/journal.pone.0183672)
Supplement: S1 Table — (PDF) [file pone.0183672.s003.pdf]

S1 Table. Quantification of behaviors of dynein on microtubules

| | | Residence time | | | fraction |
|---|---|---|---|---|---|
| Dynein only | total | 64.9 | ± | 15.6 | 100% |
| | diffusive | 50.0 | ± | 11.8 | 75% |
| | stationary | 16.9 | ± | 10.9 | 25% |
| | unidirectional | 0.0 | ± | 0.0 | 0% |
| Dynein + 1A | total | 53.2 | ± | 9.5 | 100% |
| | diffusive | 13.7 | ± | 8.2 | 25% |
| | stationary | 32.5 | ± | 11.8 | 60% |
| | unidirectional | 7.8 | ± | 5.9 | 15% |
| Dynein + 1B | total | 4.7 | ± | 3.9 | 100% |
| | diffusive | 2.7 | ± | 3.4 | 60% |
| | stationary | 1.8 | ± | 1.8 | 40% |
| | unidirectional | | N.D. | | 0% |
| Dynein + 1BΔCC1 | total | 54.0 | ± | 14.6 | 100% |
| | diffusive | 40.1 | ± | 12.4 | 71% |
| | stationary | 16.1 | ± | 5.7 | 29% |
| | unidirectional | | N.D. | | 0% |
| Dynein + p135 | total | 23.8 | ± | 3.7 | 100% |
| | diffusive | 3.8 | ± | 3.8 | 15% |
| | stationary | 20.8 | ± | 5.7 | 85% |
| | unidirectional | | N.D. | | 0% |
| Dynein + BICD2 | total | 51.8 | ± | 7.4 | 100% |
| | diffusive | 25.1 | ± | 4.9 | 50% |
| | stationary | 24.9 | ± | 6.0 | 50% |
| | unidirectional | 0.0 | ± | 0.0 | 0% |
| Dynein + 1A + BICD2 | total | 49.1 | ± | 9.5 | 100% |
| | diffusive | 5.3 | ± | 3.5 | 11% |
| | stationary | 36.3 | ± | 3.9 | 72% |
| | unidirectional | 8.8 | ± | 7.8 | 17% |
| Dynein + 1B + BICD2 | total | 19.2 | ± | 6.4 | 100% |
| | diffusive | 3.5 | ± | 2.6 | 19% |
| | stationary | 15.0 | ± | 6.2 | 81% |
| | unidirectional | | N.D. | | 0% |
| Dynein (Vi) | total | 2.2 | ± | 2.4 | 100% |
| | diffusive | 0.4 | ± | 1.3 | 17% |
| | stationary | 1.9 | ± | 2.2 | 83% |
| | unidirectional | | N.D. | | 0% |

N.D., not detected
